# Hospital Acquisition Costs for Breakthrough-Designated Devices Awarded Supplemental Medicare Reimbursement

**DOI:** 10.1001/jamahealthforum.2025.5183

**Published:** 2025-11-26

**Authors:** Osman Moneer, Kushal T. Kadakia, Sanket S. Dhruva, Joseph S. Ross, Leah M. Roth, James F. Burke, Vinay K. Rathi

**Affiliations:** 1 Department of Medicine, Stanford University, Stanford, California; 2 Massachusetts General Hospital, Boston; 3 Philip R. Lee Institute for Health Policy Studies, University of California, San Francisco; 4 Division of Cardiology, Department of Medicine, University of California, San Francisco; 5 Section of General Internal Medicine, Department of Medicine, Yale School of Medicine, New Haven, Connecticut; 6 Department of Health Policy and Management, Yale School of Public Health, New Haven, Connecticut; 7 Collaboration for Regulatory Rigor, Integrity, and Transparency, Yale School of Medicine, New Haven, Connecticut; 8 The Center for the Advancement of Team Science, Analytics, and Systems Thinking in Health Services and Implementation Science Research (CATALYST), Columbus, Ohio; 9 Department of Neurology, The Ohio State University College of Medicine, Columbus; 10 Center for Outcomes Research and Evaluation, Yale New Haven Hospital, New Haven, Connecticut; 11 Department of Otolaryngology–Head & Neck Surgery, The Ohio State University College of Medicine, Columbus

## Abstract

This cross-sectional study compares manufacturer-reported and actual hospital acquisition costs for breakthrough-designated devices awarded supplemental Medicare reimbursement.

## Introduction

Medicare bundles payment to hospitals for medical devices into prospective facility fees, which are based on historical cost estimates. To incentivize the adoption of promising, but expensive, new technologies, the Centers for Medicare & Medicaid Services (CMS) provides supplemental reimbursement through the new technology add-on payment (NTAP; inpatient) and transitional pass-through payment (TPTP; outpatient) programs.

CMS historically required devices to satisfy 3 qualifying criteria for inclusion: (1) recent (≤3 years) US Food and Drug Administration (FDA) authorization; (2) cost exceeding current reimbursement; and (3) substantial clinical improvement (vs existing alternatives). In 2020, CMS waived the substantial clinical improvement criterion for FDA breakthrough-designated devices, which have the potential to more effectively diagnose or treat serious illnesses. FDA permits greater uncertainty about breakthrough-designated device benefits and risks to expedite premarket development.^1^

CMS determines whether devices satisfy the cost criterion and establishes supplemental reimbursement amounts based on manufacturer-reported average reasonable costs at the time of application.^2^ However, hospital acquisition costs for devices are typically negotiated confidentially.^3^ We sought to compare manufacturer-reported and actual hospital acquisition costs for breakthrough-designated devices awarded supplemental reimbursement.

## Methods

We reviewed CMS Outpatient and Inpatient Prospective Payment System 2020-2024 final rules to identify all breakthrough-designated devices awarded supplemental Medicare reimbursement under NTAP/TPTP programs and manufacturer-reported costs. We limited analysis to FDA breakthrough-designated devices because (1) these technologies account for the majority of devices within NTAP^2^/TPTP programs^4^ and (2) manufacturers may price breakthrough-designated devices to optimize supplemental Medicare reimbursement.^5^ We followed STROBE reporting guidelines. Institutional review board review and the requirement for informed consent were waived per The Ohio State University College of Medicine policy on use of publicly available, nonclinical data in research without human participants.

We obtained hospital acquisition costs for breakthrough-designated devices between 2020 and 2024 using Clarivate Pricetrack, which aggregates deidentified net prices (including negotiated discounts) from hospital purchase orders; the dataset includes approximately 40% of US hospitals, with sample variation by size, location, and group purchasing organization contract (yes/no) status. We extracted device characteristics from the Devices@FDA database.

We used descriptive statistics to characterize devices and supplemental Medicare reimbursement amounts. We performed a Wilcoxon matched-pair signed rank test to examine differences between manufacturer-reported costs and purchase-volume–weighted hospital acquisition costs. We performed all analyses in R, version 4.4.7 (R Project for Statistical Computing).

## Results

Between 2020 and 2024, CMS awarded supplemental reimbursement to 26 breakthrough-designated devices, with 23 (89%) via NTAP and 10 (39%) via TPTP, including 7 (27%) qualifying for both. Hospital acquisition cost data were available for 18 devices (69%), of which 16 (90%) were therapeutic and 9 (50%) cardiovascular (Table).

**Table.** Characteristics of US Food and Drug Administration (FDA)–Designated Breakthrough Devices Included in Cost Analysis

Characteristic	No. (%)^a^
	All devices (N = 18)
FDA review pathway	
Premarket approval	10 (56)
De novo clearance	3 (17)
510(k) Clearance	5 (28)
Indication	
Diagnostic	2 (11)
Therapeutic	16 (89)
Disease area^b^	
Cardiovascular	9 (50)
Orthopedic	4 (22)
All other^c^	5 (28)
Supplemental reimbursement program^d^	
NTAP (inpatient)	16 (89)
TPTP (outpatient)	9 (50)
Maximum supplemental reimbursement per case, median (IQR), $^e^	
NTAP (inpatient)	19 750 (4963-35 000)
TPTP (outpatient)	14 094 (7035-26 625)

Abbreviations: NTAP, new technology add-on payment; TPTP, transitional pass-through payment.

^a^ Percentages may not sum to 100% due to rounding.

^b^ FDA-designated characteristic.

^c^ Including hematology, gastroenterology, physical medicine, and neurology.

^d^ NTAP and TPTP awards are nonmutually exclusive; devices may qualify for supplemental reimbursement in both inpatient and outpatient settings.

^e^ Actual payment amount varies based on hospital-specific cost data.

There was a statistically significant increase in median (IQR) manufacturer-reported costs vs purchase-volume weighted hospital acquisition costs (absolute difference, $835 [$43-$3601]; Figure, A). The median (IQR) relative difference was 9.7% (0.3%-43.6%) overall and 50% or greater for 4 devices (22%; Figure, B).

**Figure.**
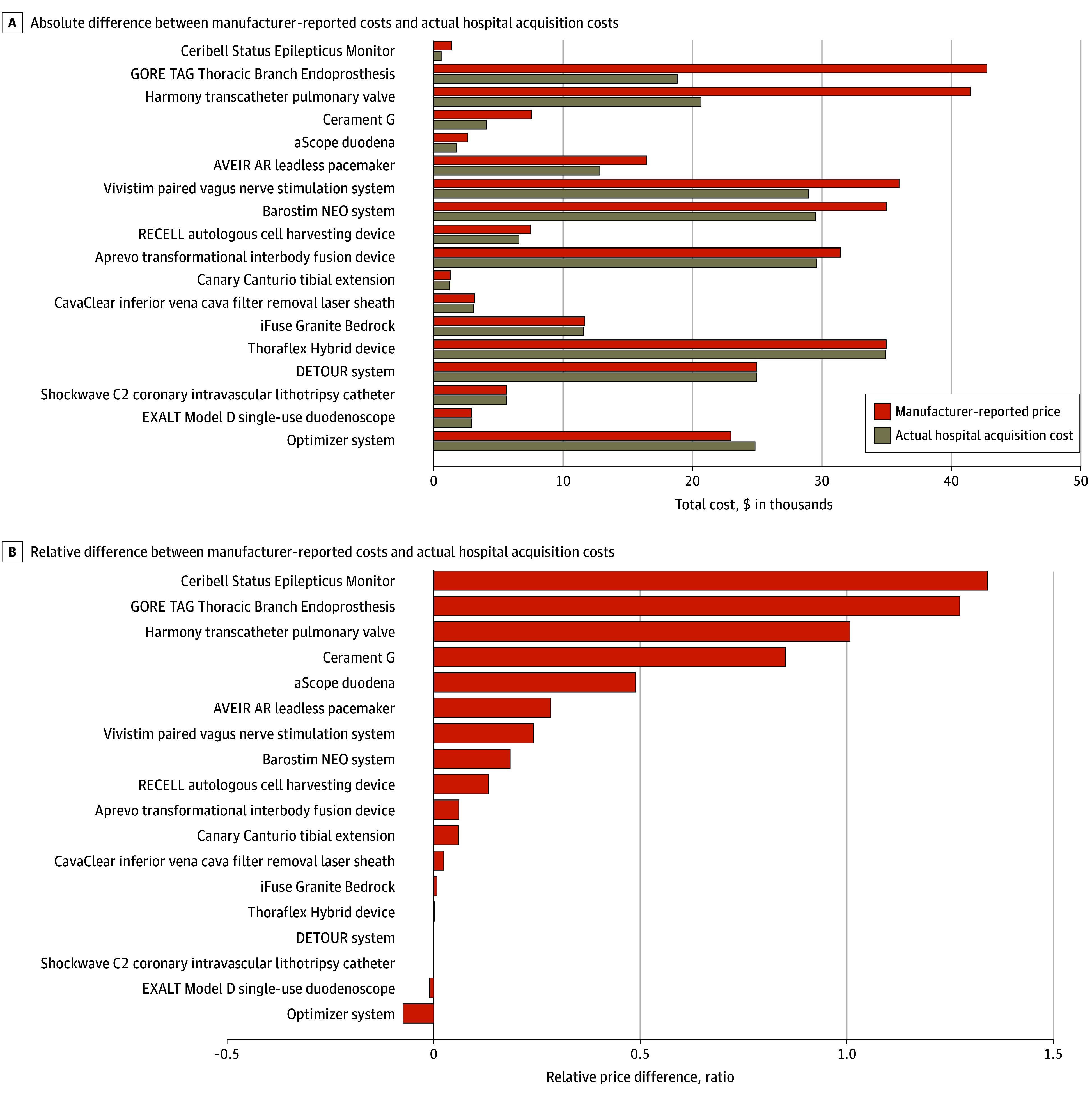
Manufacturer-Reported Costs and Actual Hospital Acquisition Costs for Medical Devices Awarded Supplemental Medicare Reimbursement, 2020-2024 Absolute costs displayed for the following 3 devices reflect manufacturer-reported estimates about case-level utilization: iFuse Bedrock Granite implant system (SI-BONE), Aprevo transforaminal IBF device (Carlsmed), and Shockwave C2 coronary intravascular lithotripsy catheter (Shockwave Medical). The Aveir AR leadless pacemaker (Abbott Cardiovascular) is not a breakthrough-designated device, but the Centers for Medicare & Medicaid Services awarded this device supplemental reimbursement as a component of a breakthrough-designated device (dual-chamber combination product with atrial and ventricular pacing).

## Discussion

In this cross-sectional study of FDA breakthrough-designated devices awarded supplemental Medicare reimbursement through the NTAP/TPTP programs, we found that manufacturer-reported costs to CMS considerably exceeded hospital acquisition costs. These findings could indicate that some manufacturers overstate device costs to trigger or augment supplemental Medicare reimbursement for hospitals.

This study has limitations. First, the results may not generalize to devices or hospitals without available hospital acquisition cost data; devices lacking these data may be less clinically or economically meaningful because utilization was not sufficient for capture through hospital purchase orders. Second, we did not evaluate changes in hospital acquisition costs over time, though the market life of included devices was short.

Recent work reveals that CMS waiver of the substantial clinical improvement criterion for breakthrough-designated devices has spurred substantial growth of the NTAP/TPTP programs.^2,4^ To limit unnecessary expenditures on these expanding programs, Congress could expand statutory requirements for manufacturers to report product average net-selling prices to CMS on a quarterly basis; these requirements already exist for Part B drugs and skin substitutes. These data would permit CMS to regularly update supplemental reimbursement eligibility and amounts based on actual hospital acquisition costs.

## Notes

Supplement.
Data Sharing Statement
