# Supplementary material for: Hospital Acquisition Costs for Breakthrough-Designated Devices Awarded Supplemental Medicare Reimbursement
Source: JAMA Health Forum. 2025 Nov 26;6(11):e255183. doi: 10.1001/jamahealthforum.2025.5183 (PMC12658659; doi:10.1001/jamahealthforum.2025.5183)
Supplement: Supplement. — Data Sharing Statement [file jamahealthforum-e255183-s001.pdf]

Data Sharing Statement

Moneer. Hospital Acquisition Costs for Breakthrough-Designated Devices Awarded Supplemental Medicare Reimbursement. *JAMA Health Forum*. Published November 26, 2025. doi:10.1001/jamahealthforum.2025.5183

Data
Data available: No

Additional Information
Explanation for why data not available: We obtained hospital acquisition costs using Clarivate Pricetrack, a proprietary dataset.
